# Effects of *Gelidium elegans* on Weight and Fat Mass Reduction and Obesity Biomarkers in Overweight or Obese Adults: A Randomized Double-Blinded Study

**DOI:** 10.3390/nu11071513

**Published:** 2019-07-03

**Authors:** Choon Ok Kim, Youn Nam Kim, Duk-Chul Lee

**Affiliations:** 1 Department of Clinical Pharmacology and Clinical Trials Center, Severance Hospital, Yonsei University Health System, Seoul 03722, Korea; 2 Clinical Trials Center, Severance Hospital, Yonsei University Health System, Seoul 03722, Korea; 3 Department of Family Medicine, Yonsei University, College of Medicine, Seoul 03722, Korea

**Keywords:** *Gelidium elegans*, obese, overweight, weight, fat mass

## Abstract

The edible seaweed *Gelidium elegans* (GEE) is known to inhibit adipocyte differentiation. However, there has been no report on its effects in humans. In this study, we investigated whether GEE reduces body weight or fat mass in obese or overweight individuals. A total of 78 participants were randomly assigned to the test (GEE extract 1000 mg/day) and placebo groups at a 1:1 ratio, and treated for 12 weeks. At six or 12 weeks after randomization, they were evaluated for anthropometric parameters, biomarkers, and body composition. Changes in body weight and fat mass between the two groups was significantly different, as determined using ANCOVA adjusted for baseline, calorie intake, and physical activity. Body weight and fat mass were significantly decreased by GEE after 12 weeks but increased in the placebo group. Moreover, although not significant, triglyceride levels tended to decrease after GEE intake. There was no significant difference in other laboratory biomarkers between the two groups. Taken together, these results suggested that GEE significantly reduced body weight, especially fat mass, in overweight or obese individuals.

## 1. Introduction

The population of obese individuals has been rapidly growing worldwide. Approximately 38% of the adult population in the world is estimated to be overweight and another 20% will be obese by 2030 [1]. Obesity is quite simply defined as excessive fat accumulation; however, it is the most important risk factor for major chronic diseases, including hypertension, diabetes mellitus, cardiovascular disease, musculoskeletal disease, depression, and cancer [2,3]. Since the annual healthcare burden attributable to obesity and obesity-related disease has increased [3], there have been increased efforts to decrease the prevalence of obesity and to treat or prevent it to reduce the burden of obesity-related co-morbidities and improve the overall health-related quality of life [4,5]. 

A few FDA-approved drugs are currently available for long-term management of obesity, namely phentermine-topiramate extended release, lorcaserin, bupropion-naltrexone, orlistat, and liraglutide [5]. These medications are approved only for patients with a body mass index (BMI) of ≥30 kg/m^2^ or ≥27 kg/m^2^, with at least one obesity-related co-morbidity [6,7]. Despite pharmacotherapy having an effect on weight loss in the range of 5%–15%, 63.4% of patients fail to sustain a 5% weight loss in the long term [8,9]. Weight is typically regained when medication is stopped [5]. Therefore, lifestyle modifications, including adequate diet and physical activity, must accompany therapy to maintain desirable weight loss and prevent excessive weight gain.

In general, low-calorie diets with low glycemic index are recommended, and intake of high quality foods, such as fruits and vegetables, is vital [6,10]. In addition, extensive research has been ongoing to identify healthy foods that can prevent excessive weight gain [11,12,13]. Some studies have shown that the edible seaweed *Gelidium elegans* (GEE), red alga, has nutraceutical activities, such as anti-adipogenesis and anti-obesity effects in vivo [11,14,15]. High-fat diet-fed mice administered GEE exhibited less weight gain and lower amounts of subcutaneous and abdominal fat than high-fat diet-fed mice not treated with GEE [11,14,15]. However, to date, no study has been conducted on humans to explore any possibility of a preventive effect of GEE on weight gain, as in the mouse model of obesity. In this study, we investigated whether GEE intake reduces body weight or fat accumulation in obese or overweight patients.

## 2. Materials and Methods 

### 2.1. Ethics

The study protocol was approved by the institutional review board of Severance hospital (Seoul, Republic of Korea, IRB number 4-2017-0860). It is also registered at the ClinicalTrials.gov (Identifier: NCT03842774). This study was performed in accordance with the Declaration of Helsinki and Korean Good Clinical Practice (KGCP) guidelines. All subjects provided written informed consent prior to enrolment in the study.

### 2.2. Participants

We recruited 109 volunteers from Severance Hospital, Seoul, Republic of Korea. Subjects aged between 19 and 50 years, with BMI between 23.0 and 30.0 kg/m^2^, were considered eligible for this study. In addition, subjects who met the following criteria were excluded: (1) Patients presenting clinically significant cerebrovascular and cardiovascular disease, arrhythmia, congestive heart failure, cancer, psychiatric disorders, or hepatic or renal disease; (2) patients with uncontrolled hypertension (systolic blood pressure ≥160 mmHg or diastolic blood pressure ≥100 mmHg), diabetes mellitus, or thyroid disease; (3) a night worker or shift worker; (4) patients currently prescribed anti-obesity, thyroid hormone, steroid, diuretic, or female sex hormone medications; (5) patients who experienced weight loss within three months prior to the start of the study; (6) patients with abnormal laboratory results (creatinine, aspartate aminotransferase, or alanine aminotransferase level ≥2.0 ×upper normal limit).

### 2.3. Study Design and Intervention

**Figure 1:**
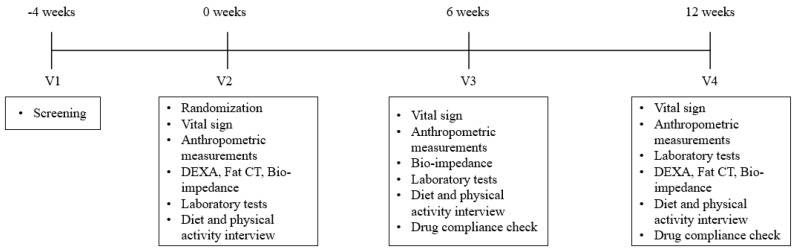
Overview of the study design.

This was a randomized, double-blinded, multiple-dose, parallel study; the overall study schedule is shown in Figure 1. All subjects registered in this study underwent the following baseline evaluation: Blood pressure, anthropometric measurement, diet and physical activity assessment, laboratory tests including lipid profile, body composition using bioelectrical impedance analyzer (InBody U20, Biospace, Seoul, Republic of Korea), dual energy X-ray absorptiometry (DEXA; BHR-140-P Discovery A, Hologic Inc., Bedford, MA, USA), and fat-measurement computed tomography (fat CT; Tomoscan 350, Philips, Mahwah, NJ, USA). After baseline evaluation, the participants were randomly assigned to the test and placebo groups at a 1:1 ratio. The subjects of the test group received three tablets of GEE extract (1000 mg/day) once a day for 12 weeks, and those of the placebo group received placebo for 12 weeks in the same regimen as the test group. Placebo tablets had similar color, flavor, and form as those of the GEE extract. GEE extract and placebo tablets were supplied by NEWTREE (Seongnam, Kyonggi, Republic of Korea). GEE extract were prepared using the following method. GEE was collected in Jeju Island, Republic of Korea. The collected wild GEE were washed with water and extracted with 70% ethanol for overnight. The supernatants of the extracts were filtered through a 50-μm bag paper and then concentrated at a temperature below 80 °C. The precipitants were extracted with water at 90 °C for 2 h, and then filtered and concentrated in the same manner. The two extracts were mixed and heated at 90 °C for 20 min, and then filtered through a 50-μm bag filter. Subsequently, they were dried using a spray dryer.

Participants visited the clinic for evaluation of the effects of the study treatment (GEE extract or placebo) at six and 12 weeks after randomization. At six and 12 weeks, blood pressure, as well as waist and hip circumferences were measured, and the patients were assessed by bioelectrical impedance analyzer. They were also subjected to DEXA and fat CT for assessment of body composition at 12 weeks. At every visit, they were asked about the first and last date of GEE extract or placebo treatment administration, and the remaining treatment tablets provided during the previous visit were returned. In addition, diet and physical activity were evaluated. During the study period, all subjects were prohibited from receiving any diuretic, steroid, antidiabetic, psychiatric, anti-obesity, female sex hormone, thyroid hormone, herbal, and over-the-counter medications and supplements, which could affect body weight or body composition evaluation. 

### 2.4. Anthropometric Measurement and Body Composition

For screening tests (visit 1), we measured body weight (kg) and height (cm), approximated to the first decimal, using an automatic extensometer (BSM 330; Biospace, Seoul, Republic of Korea) while the participants were wearing light clothes, and BMI was calculated as the ratio of weight (kg) to height^2^ (m^2^). From visit 2 onwards, both body weight and BMI were assessed by the bioelectrical impedance analyzer, and the values were used for evaluation of treatment effects. Waist circumference (cm) was measured midway between the bottom edge of the last rib and the iliac crest in the mid-axillary plane. Hip circumference (cm) was measured at the level of the widest circumference over the buttocks. Waist–hip ratio was calculated as the ratio of waist-to-hip circumference (cm). 

To evaluate body composition, we used a bioelectrical impedance analyzer, DEXA, and fat CT. Bioelectrical impedance analyzer measured fat mass, fat percentage, and skeletal muscle content. DEXA was used to assess total body mass, total fat mass, and total lean body mass. Fat CT scan, sliced by 10-mm, was used to evaluate subcutaneous and visceral fat areas, measured at the level of L3–L4, since it represents the limit of the upper abdomen and is not influenced by liver or adipose tissue from the buttocks [16]. 

### 2.5. Blood Collection and Analysis

To assess the effect of treatment on metabolic parameters, blood samples were collected after a 10-h overnight fasting between visit 2 and 4. White blood counts were quantified using a XN-9000 Hematology Analyzer (Sysmex, Kobe, Japan). Fasting glucose, high-sensitivity C-reactive protein (hs-CRP), total cholesterol, triglyceride content, high-density lipoprotein (HDL), and low-density lipoprotein (LDL) cholesterol levels were measured with an ADVIA 1650 Clinical Chemistry System (Siemens Medical Solutions, Tarrytown, NY, USA). Fasting insulin level was measured by an electrochemiluminescence immunoassay using an Elecsys 2010 instrument (Roche, Indianapolis, IN, USA). Insulin resistance and β-cell function were estimated using the homeostasis model analysis (HOMA) method, by applying the following formula: HOMA-insulin resistance (IR) = {fasting insulin (μIU/mL) × fasting glucose (mmol/L)}/22.5; HOMA- β = {20 × fasting insulin (μIU/mL)}/{fasting glucose (mmol/L) – 3.5} [17]. 

### 2.6. Dietary and Physical Activity Assessment

Participants were provided with detailed instructions on how to record their diet and physical activity by a designated and well-trained physician prior to the study, and they were asked to maintain the same lifestyle throughout the study period. Moreover, they were prohibited from consuming any food containing GEE during the study period. To assess food consumption at baseline and follow-up visits, participants were asked to complete three-day diet records of all food and beverage intake over a total of three days in the week immediately preceding the next visit. One of the three days could include a weekend day. Data from the three-day diet records were analyzed using a computer-aided nutritional analysis program CANPRO 3.0 (APAC Intelligence, Seoul, Republic of Korea) to evaluate the total calorie intake [18].

To assess physical activity, we used the International Physical Activity Questionnaire-Short Form (IPAQ-SF) at baseline and follow-up visits [19]. The IPAQ-SF records the last seven-day report of four intensity levels of physical activity, including vigorous-intensity activity, moderate-intensity activity, walking, and sitting. The IPAQ-SF data were converted to metabolic equivalent minutes per week (MET-min/week) using the published formulation [20]. Compendium of average MET score was derived for each type of activity: Walking = 3.3 METs; moderate-intensity activity = 4.0 METs; and vigorous-intensity activity = 8.0 METs [20]. Using these values, activities corresponding to each intensity were defined as continuous values; MET-min/week in each intensity activity = (average MET score) × (average minutes in a day) × (average days in a week) in each intensity activity [19].

### 2.7. Statistical Analysis

The data were analyzed using the SAS statistical software version 9.3 (SAS Institute Inc. Cary, NC, USA). The efficacy and baseline-analysis set included randomized participants who were compliant with the study protocol and received study treatments of at least >80% during the study. Baseline characteristics between the two treatment groups were compared using independent two sample *t*-tests or Wilcoxon rank sum test for continuous data and chi-square test for categorical data. Difference in parameters between the two groups after intervention was analyzed by an independent two sample *t*-test or Wilcoxon rank sum test, and within-group differences were analyzed by paired *t*-test or Wilcoxon signed rank test. We actually planned to analyze t-tests but, in case of rejecting in the Shapiro–Wilk normality test, and then Wilcoxon tests were used to compare their differences. In addition, changes from baseline of each parameter between the two groups were analyzed using analysis of covariance (ANCOVA), which was adjusted for its baseline value, total calories, and intensity of physical activity (MET-min/week). In an ANCOVA test, the adjusted values of parameters were used directly. The changes from baseline of total calories and physical activity were calculated as follows: Δ6 week (or 12 week) = {values at 6 weeks (or 12 weeks)} – {baseline}. The total calories and physical activity used for the ANCOVA test were the average of the values evaluated at six and 12 weeks. All data were expressed as mean ± standard deviation (SD), median (interquartile range), or number (%). The statistical tests were two-sided, and statistical significance was defined as *p* < 0.05. The purpose of this study was not confirmatory, but exploratory; therefore, we did not consider any correction for the multiple comparison.

## 3. Results

**Figure 2:**
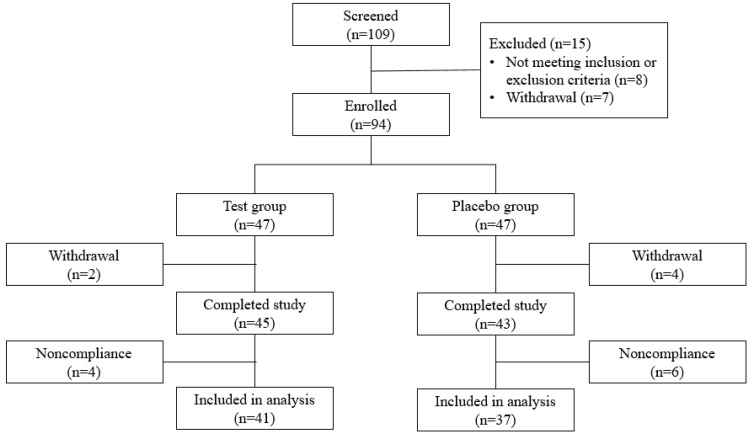
Flowchart illustrating the inclusion and exclusion of the study subjects.

A total of 94 subjects were enrolled in this study and randomized into the test or placebo group at a ratio 1:1 (Figure 2). Two subjects in the test group and four subjects in the placebo group withdrew voluntarily from the study. A total of 88 subjects completed the study; however, 10 subjects with <80% treatment compliance during the study were excluded from the analysis. Therefore, 41 subjects in the test group and 37 subjects in the placebo group were included in data analysis. 

### 3.1. Baseline Characteristics

The baseline characteristics of each treatment group are presented in Table 1. No significant differences between the two groups were observed with respect to age, sex, anthropometric measurements, blood pressure, lipid profiles, calorie intake, and physical activity.

### 3.2. Diet and Physical Activity 

Table 2 shows energy intake and physical activity for each group throughout the study period. Compared with baseline, calorie intake after 12 weeks decreased in the GEE group, but was similar in the placebo group. However, there were no significant differences in total calories at six and 12 weeks after the study (*p >* 0.05 by Wilcoxon rank sum test). In addition, the change in calorie intake after six and 12 weeks was not significantly different between the test and placebo groups (*p >* 0.05 by Wilcoxon rank sum test). 

Although there were no significant differences in physical activity between the two groups at six and 12 weeks after the study (*p >* 0.05 by Wilcoxon rank sum test), the change in each group was different from baseline. The placebo group maintained similar activity with baseline; however, vigorous and walking activity in the GEE group increased compared to baseline (*p <* 0.05 by Wilcoxon signed rank test). Moreover, there was a significant difference in the changes from baseline after 12 weeks in total physical activity between the two groups (*p <* 0.05 by Wilcoxon rank sum test).

### 3.3. Anthropometrics and Body Composition

**Figure 3:**
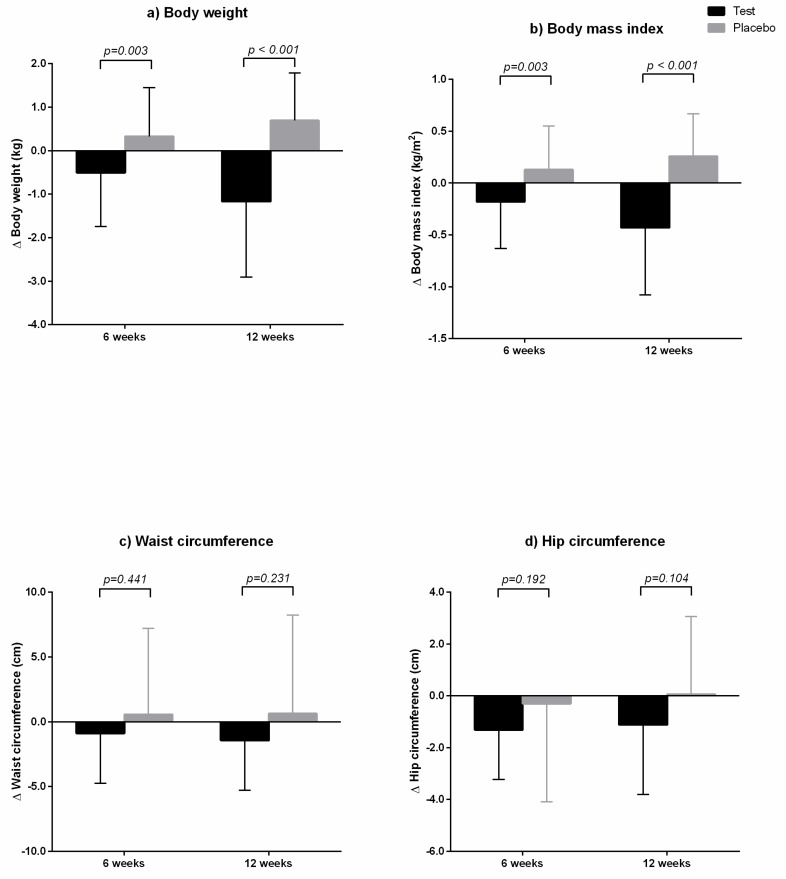
Changes in anthropometric parameters from baseline between the two groups at six and 12 weeks. The test group was administered *Gelidium elegans* extract 1000 mg/day for 12 weeks, and the placebo group was administered placebo. (**a**) Body weight; (**b**) body mass index; (**c**) waist circumference; and (**d**) hip circumference. *p*-values were calculated using ANCOVA test adjusted for baseline value, calorie intake, and physical activity.

After six and 12 weeks of GEE intake, body weight and BMI decreased compared to baseline values, but those in the placebo group increased compared to baseline values (Table 3). There were no statistically significant differences in body weight and BMI between the two groups at each time point (*p* > 0.05 by independent t-test); however, changes from baseline between the two groups at six and 12 weeks were significantly different, as analyzed by ANCOVA test adjusted for baseline value, calorie intake, and physical activity (Figure 3). After 12 weeks of GEE intake, waist circumference and hip circumference decreased significantly; however, the differences were not statistically significant compared to those in the placebo group, as determined by ANCOVA test.

**Figure 4:**
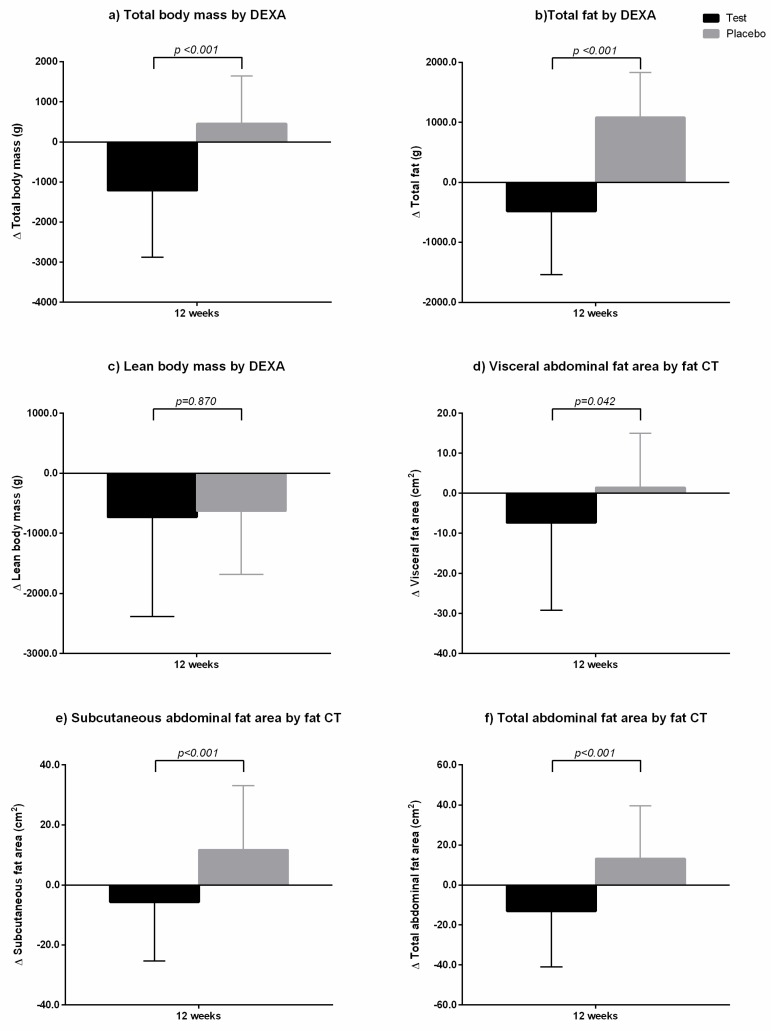
Changes in body composition from baseline between the two groups at 12 weeks. The test group was administered *Gelidium elegans* extract 1000 mg/day for 12 weeks, and the placebo group was administered placebo in a similar manner. (**a**) Total body mass by DEXA; (**b**) total fat mass by DEXA; (**c**) lean body mass by DEXA; (**d**) visceral abdominal fat area by fat computed tomography (CT); (**e**) subcutaneous abdominal fat area by fat CT; and (**f**) total abdominal fat area by fat CT. *p*-values were calculated using an ANCOVA test adjusted for baseline value, calorie intake, and physical activity.

Table 4 and Figure 4 show changes in body composition, as measured by DEXA. At baseline, total fat mass in the two groups was similar with no significant difference (*p* = 0.919). After 12 weeks, total fat mass in the test group decreased compared to baseline value, but that in the placebo group increased compared to the baseline value. Total body mass showed similar pattern as that of total fat mass change, although lean body mass decreased in both groups. GEE or placebo administration once a day for 12 weeks decreased total fat mass in the test group to 0.48 ± 1.06 kg compared to baseline, whereas total fat mass in the placebo group increased to 1.08 ± 0.75 kg compared to baseline (Figure 4). These changes between the two groups were statistically significant, as revealed by ANCOVA test adjusted for baseline value, calorie intake, and physical activity. In addition, we analyzed the body composition measured by DEXA according to gender. There were the same results with those that were shown in Table 4. In both male and female, the total body mass and fat mass decreased in the GEE group while those increased in the placebo group (data not shown).

Abdominal fat area measured by fat CT is shown in Table 4 and Figure 4. At baseline, there were no significant differences in visceral fat area (VFA), subcutaneous fat area (SFA), and total abdominal fat area (TAF; *p* > 0.05 by an independent *t*-test). After 12 weeks of GEE or placebo administration, VFA, SFA, and TAF in the test group decreased to 7.36 ± 21.83 cm^2^, 5.67 ± 19.68 cm^2^, and 13.03 ± 27.95 cm^2^, respectively, compared to baseline values, whereas those in the placebo group increased to 1.45 ± 13.53 cm^2^, 11.63 ± 21.49 cm^2^, and 13.08 ± 26.51 cm^2^, respectively, compared to baseline values (Figure 4). The abdominal fat areas between the two groups at 12 weeks were not significantly different, as analyzed by independent test; however, the changes from baseline between the two groups were statistically significant, as determined by ANCOVA test adjusted for baseline value, calorie intake, and physical activity. 

### 3.4. Laboratory Parameters

**Figure 5:**
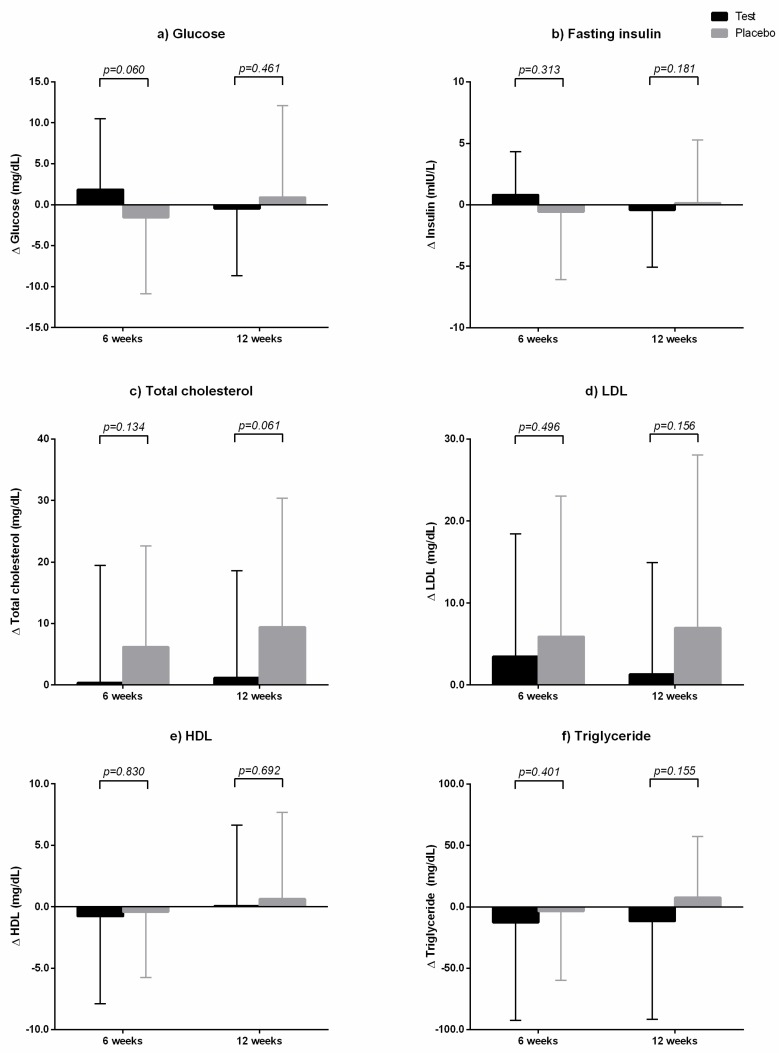
Laboratory changes from baseline between the two groups at six and 12 weeks. The test group was administered *Gelidium elegans* extract 1000 mg/day for 12 weeks, and the placebo group was administered placebo in a similar manner. (**a**) Glucose; (**b**) fating insulin; (**c**) total cholesterol; (**d**) LDL; (**e**) HDL; and (**f**) triglyceride. *p*-values were calculated using an ANCOVA test adjusted for baseline value, calorie intake, and physical activity.

Although fasting glucose, fasting insulin, triglyceride levels increased in the placebo group, they decreased after 12 weeks in the GEE group. These results were consistent with body weight changes, which were observed in two groups. However, these changes within-group did not have statistical significance. In addition, after 12 weeks of treatment with GEE or placebo, there were no significant differences in fasting glucose, fasting insulin, HOMA-IR, HOMA-β, and hs-CRP levels between the two groups (Table 5). The changes in the above parameters from baseline between the two groups were also not significantly different (Figure 5).

After 12 weeks, total cholesterol and LDL in the test group were similar to their baseline levels, whereas triglyceride level decreased to 11.66 ± 79.86 mg/dL compared to baseline value (Table 5 and Figure 5). In the placebo group, total cholesterol, LDL, and triglyceride levels increased to 9.43 ± 20.94 mg/dL, 7.00 ± 21.06 mg/dL, and 7.49 ± 49.92 mg/dL, respectively, compared to baseline values. However, the above parameters did not significantly differ between the two groups at each time point, as revealed by independent *t*-tests (Table 5). The changes in lipid parameters, including triglycerides, from baseline between the two groups were also not statistically significant, as analyzed by an ANCOVA test adjusted for baseline value, calorie intake, and physical activity (Figure 5).

## 4. Discussion

This randomized, double-blinded study was conducted to investigate the effects of GEE on body composition and obesity-related biomarkers in overweight or obese participants. We observed that participants administered GEE for 12 weeks showed a significant decrease in body weight and BMI compared to the placebo group. In particular, the GEE group showed a decrease in total body fat mass, including visceral abdominal fat. In addition, although not significant, the triglyceride level decreased after GEE intake. 

Excess energy intake relative to energy expenditure leads to an increase in adipocyte number and volume, which, in turn, increases adipose tissue mass [21]. This adipogenesis can be divided into three main phases: Determination, clonal expansion, and terminal differentiation [22]. It is tightly regulated by a cascade of transcriptional factors [22]. Adipogenic stimuli, such as hormones, growth factors, and cytokines, induce CAAT/enhancer-binding protein β (C/EBPβ) and C/EBPδ [23]. These proteins subsequently induce the expression of peroxisome proliferator-activated receptor gamma (PPARγ) and C/EBPα, which are the key transcriptional factors for adipogenesis [23]. Sterol regulatory element-binding transcription factor-1 (SREBP-1) is also an important transcription factor that promotes early adipocyte differentiation, facilitates fatty acid metabolism, and might induce the expression of PPARγ [23].

Several studies have reported that GEE inhibits the process of adipocyte differentiation. An in vitro study showed that the treatment of 3T3-L1 cells with GEE increases PPARγ and C/EBPα expression and decreases lipid accumulation in a dose-dependent manner [24]. These effects were initially considered to be due to the flavonoids contained in GEE, such as rutin and hesperidin, which were known to have anti-adipogenic activity [22,25]. However, similar results were noted for a low concentration of GEE without flavonoids in an in vitro study [26]. This suggests that GEE may suppress adipogenesis independently of flavonoids.

Similar results were observed in animal studies wherein GEE inhibits adipocyte differentiation and lipid accumulation [11,14,15]. Mice were maintained on a high-fat diet, and one group was administered GEE whereas the other group was not. After a few weeks, the mice fed high-fat diet alone showed a continuous increase in body weight, whereas the mice fed high-fat diet along with GEE showed suppressed weight gain, and the effect increased as the GEE dose increased. In addition, the mice administered GEE showed relatively low accumulation of subcutaneous and abdominal fat, compared to the mice not administered GEE. The expression levels of transcription factors in adipose tissue were evaluated to elucidate the molecular mechanism of GEE in mice, and the results showed that levels of PPARγ, C/EBPα, and SREBP-1 in the GEE-supplemented group were significantly decreased compared to those in the high-fat diet alone group [11,14,15]. 

Our study showed that GEE administration for 12 weeks decreased body weight and total fat mass. These results were similar to those of in vivo studies. In this study, we did not evaluate the molecular mechanism of GEE in humans. However, on the basis of results of in vivo and in vitro studies, GEE could inhibit adipocyte differentiation in humans by suppressing PPARγ and C/EBPα, and prevent fat accumulation and weight gain. Although this study has limitations in explaining the mechanism of GEE in regulating adipogenesis in humans, it was the first study to investigate the effects of GEE in humans. Additionally, unlike animal studies involving use of a high-fat diet, in this study, we encouraged participants to maintain their calorie intake similar to baseline values, and there were no differences in energy intake between the two groups during the study. This suggested that GEE supplementation with a regular diet might exert a similar inhibitory effect on adipogenesis. 

We educated the subjects to maintain the same level of physical activity during the study as that at baseline, and we checked physical activity at each visit. However, in the placebo group, physical activity was similar or decreased, whereas in the GEE group, physical activity was increased compared to the baseline. Although a well-trained researcher consistently educated the participants, this study has a limitation that physical activity was not controlled at the same level as the baseline, which could have affected body weight. However, since this was a randomized, controlled, double-blinded study, the bias of the researchers and subjects in lifestyle management were well controlled. Moreover, considering that caloric intake after 12 weeks was similar between the two groups, the increase in physical activity in the GEE group was likely to have been caused by chance. Since an increase in physical activity is closely related to body weight loss, we conducted an ANCOVA test with adjustment for variables that might affect body weight, including physical activity. In the results of the ANCOVA test adjusted for the baseline value of each parameter, calorie intake, and physical activity, the GEE group showed a significant decrease in body weight and body fat compared to the placebo group. Therefore, our findings suggested that GEE significantly reduced body weight and body fat.

There was no significant difference in fasting glucose level and lipid profiles between the GEE and placebo groups after 12 weeks of each treatment. However, considering that the participants’ characteristics were within the normal range at baseline, these changes could be meaningful. The laboratory baseline values in Table 5 indicated that the average fasting glucose level, fasting insulin level, and lipid profiles in this study were in the normal range. After 12 weeks of treatment with GEE or placebo, these parameters were decreased in the GEE group whereas not all parameters were increased in the placebo group. That is, the laboratory changes observed in the GEE group were consistent with the result of weight loss after 12 weeks. Therefore, we suggest that those changes in laboratory parameters were clinically significant considering that the participants were near-healthy subjects and the parameters were within the normal range at baseline.

The main feature of fatty liver disease is triglyceride accumulation in the cytoplasm of hepatocytes [27]. Non-esterified fatty acids in plasma are transported into the cytoplasm of hepatocytes and then rapidly activated through conversion to fatty acyl-CoAs [27]. Fatty acids are also synthesized de novo by acetyl-CoA carboxylase (ACC) and fatty acid synthase (FAS) in the liver [27]. Fatty acids in the hepatocytes are then converted to triglycerides by various factors, such as stearoyl-CoA desaturases 1 (SCD1), lipin 1, and acyl-CoA:diacylglycerol acyltransferase (DGAT) [27]. In this study, triglyceride level in the GEE groups decreased although not significantly after 12 weeks. It is difficult to claim that GEE prevented fatty liver disease in humans because we did not evaluate triglyceride accumulation in hepatocytes. However, approximately 60% of hepatic triglycerides in humans is obtained from non-esterified fatty acids in plasma [28]. Plasma non-esterified fatty acids are elevated in fatty liver disease and obese subjects [27]. Fatty liver disease is strongly associated with obesity, and increased fat mass contributes directly to greater fatty acid release from adipose tissue [27]. In animal studies to investigate the effects of GEE on hepatic lipogenesis, mice with GEE showed decreased liver weight and liver triglyceride content [11,14,15]. In high-fat diet-induced obese mice, GEE decreased the expression of SREBP-1, ACC, FAS, and DGAT-1 [11,14]. SREBP-1 is a transcription factor that promotes the expression of lipogenic genes, including FAS, ACC, SCD1, and lipin 1 [29,30]. Moreover, GEE induced the expression of thermogenesis regulatory molecules, such as adenosine monophosphate-activated protein kinase, PR domain-containing 16, and uncoupling protein-1 in hepatic tissues, indicating that GEE may increase energy metabolism [11]. Considering these results from animal studies, the reduction of triglycerides and fat mass in the GEE group of this study might be an indication that, in humans, GEE could play the role of a negative regulator of lipogenesis and might inhibit the accumulation of triglycerides in hepatocytes. Further studies in patients with fatty liver disease are needed to evaluate whether GEE prevents triglyceride accumulation in hepatocytes or improves fatty liver disease severity.

## 5. Conclusions

In conclusion, our findings indicated that GEE intake might have a beneficial effect in ameliorating body weight, total fat mass, subcutaneous fat, visceral fat, and triglycerides in overweight and obese individuals regardless of whether these individuals change their lifestyle to reduce body weight. On the basis of the mechanism of GEE with respect to adipocyte differentiation and hepatic lipogenesis, the results of this study suggested that GEE supplementation might reduce body weight, especially body fat mass, and its effect could be expanded to reduce hepatic fat accumulation. The efficacy of GEE against body fat will be useful considering that the size of the obese population has been increasing but anti-obesity treatment is limited. However, to confirm the efficacy of GEE against obesity or fat accumulation, further studies are necessary to investigate whether these effects can be obtained even over long-term administration and whether GEE has an effect on fatty liver disease.

## Figures and Tables

**Table 1:** Demographic and baseline characteristics of the study population.

Demographic Variables	Test (*n* = 41)	Placebo (*n* = 37)	*p*-Value
Age (years)	36.66 ± 8.55	34.22 ± 6.89	0.172
Sex			
Male	17 (41.5)	14 (37.8)	0.924
Body weight (kg)	72.11 ± 11.88	70.87 ± 10.56	0.629
Body mass index (kg/m^2^)	25.78 ± 1.95	25.85 ± 1.92	0.863
Waist circumference (cm)	89.29 ± 7.84	87.29 ± 10.07	0.328
Hip circumference (cm)	102.59 ± 5.03	101.71 ± 4.83	0.438
Waist-to-hip ratio	0.87 ± 0.05	0.86 ± 0.09	0.493
SBP (mmHg)	125.85 ± 13.71	124.73 ± 13.71	0.734
DBP (mmHg)	77.56 ± 8.92	75.22 ± 11.77	0.322
Fasting glucose (mg/dL)	93.49 ± 10.38	93.49 ± 9.51	0.9995
Fasting insulin (uIU/mL)	8.45 ± 4.93	9.51 ± 5.62	0.377
HOMA-IR	1.98 ± 1.25	2.24 ± 1.45	0.396
HOMA-β	94.52 ± 94.34	120.53 ± 70.79	0.176
hs-CRP (mg/dL)	1.36 ± 1.96	2.14 ± 5.79	0.442
Total cholesterol (mg/dL)	190.37 ± 28.98	190.03 ± 36.68	0.964
LDL (mg/dL)	112.95 ± 27.99	112.81 ± 34.57	0.985
HDL (mg/dL)	56.59 ± 10.97	57.19 ± 12.51	0.821
Triglyceride (mg/dL)	125.22 ± 123.37	121.81 ± 83.08	0.886
AST (IU/L)	19.00 (6.00)	17.00 (4.00)	0.022
ALT (IU/L)	18.00 (10.00)	15.00 (8.00)	0.045
BUN (mg/dL)	12.20 ± 3.21	12.56 ± 2.55	0.583
Creatinine (mg/dL)	0.74 ± 0.17	0.71 ± 0.17	0.471
TSH (mIU/mL)	1.50 ± 1.29	1.55 ± 0.75	0.828
Calorie (kcal/day)	1715.64 ± 507.85	1737.11 ± 449.70	0.845
Physical activity (MET-min/week)	1696.71 ± 2204.96	2981.16 ± 3601.92	0.066

Data are presented as mean ± standard deviation except for sex, which is expressed as *n* (%), and AST and ALT, which is expressed as median (interquartile range). *p*-values were calculated using independent two sample *t*-test for continuous variables except for AST and ALT and chi-square test for categorical variables. AST and ALT were analyzed by Wilcoxon rank sum test. SBP, systolic blood pressure; DBP, diastolic blood pressure; HOMA-IR, homeostasis model analysis-insulin resistance; hs-CRP, high-sensitivity C-reactive protein; LDL, low-density lipoprotein; HDL, high-density lipoprotein; AST, aspartate aminotransferase; ALT, alanine aminotransferase; BUN, blood urea nitrogen; TSH, thyroid stimulating hormone.

**Table 2:** Energy intake and physical activity.

Variables		Test (*n* = 41)	Placebo (*n* = 37)	*p*-Value *
**Calories (kcal/day)**				
	Baseline	1687 (685)	1728 (623)	0.777
	6 weeks	1577 (336)	1676 (391)	0.495
	12 weeks	1597 (521)	1732 (732)	0.255
	Δ_6 week-baseline_	−94 (522)	−106 (537)	0.948
	Δ_12 week-baseline_	−63 (853)	17 (653)	0.508
**Physical activity (MET-min/week)**				
Vigorous	Baseline	0 (720)	0 (720)	0.923
	6 weeks	0 (800)	0 (720)	0.839
	12 weeks	0 (1200)	0 (720)	0.577
	Δ_6 week-baseline_	0 (240)	0 (0)	0.216
	Δ_12 week-baseline_	0 (240) **	0 (320)	0.043
Moderate	Baseline	0 (360)	0 (240)	0.978
	6 weeks	0 (480)	0 (360)	0.848
	12 weeks	0 (360)	0 (320)	0.342
	Δ_6 week-baseline_	0 (360)	0 (120)	0.881
	Δ_12 week-baseline_	0 (360)	0 (160)	0.436
Walking	Baseline	495 (7590)	693 (924)	0.027
	6 weeks	594 (4158)	693 (1320)	0.157
	12 weeks	792 (1056)	924 (1386)	0.344
	Δ_6 week-baseline_	0 (561)	198 (957)	0.753
	Δ_12 week-baseline_	0 (660) **	0 (462)	0.146
Total	Baseline	990 (1941)	1746 (3465)	0.080
	6 weeks	1386 (1839)	1386 (2493)	0.473
	12 weeks	1386 (2319)	1710 (2136)	0.625
	Δ_6 week-baseline_	141 (1287)	247 (2331)	0.705
	Δ_12 week-baseline_	240 (1761) **	–9 (1062)	0.043

Data are presented as the median (interquartile range). * *p*-values were calculated using Wilcoxon rank sum test. ** were represented as having *p*-values <0.05 in the Wilcoxon signed rank test for comparison within-group differences.

**Table 3:** Effect of *Gelidium elegans* on anthropometric parameters.

Variables		Test (*n* = 41)	Placebo (*n* = 37)	*p*-Value *
Body weight (kg)	Baseline	72.11 ± 11.88	70.87 ± 10.56	0.629
	6 weeks	71.60 ± 11.72	71.20 ± 10.34	0.873
	12 weeks	70.94 ± 11.88	71.56 ± 10.38	0.807
	Δ_6 week-baseline_	−0.51 ± 1.23 **	0.33 ± 1.12	0.002
	Δ_12 week-baseline_	−1.17 ± 1.74 **	0.69 ± 1.10**	<0.0001
Body mass index (kg/m^2^)	Baseline	25.78 ± 1.95	25.85 ± 1.92	0.863
	6 weeks	25.60 ± 1.92	25.98 ± 1.95	0.387
	12 weeks	25.35 ± 1.97	26.11 ± 1.96	0.090
	Δ_6 week-baseline_	−0.18 ± 0.45 **	0.13 ± 0.42	0.003
	Δ_12 week-baseline_	−0.43 ± 0.65 **	0.26 ± 0.41 **	<0.0001
Waist circumference (cm)	Baseline	89.29 ± 7.84	87.29 ± 10.07	0.328
	6 weeks	88.41 ± 7.79	87.85 ± 7.18	0.742
	12 weeks	87.85 ± 8.56	87.91 ± 8.33	0.974
	Δ_6 week-baseline_	−0.88 ± 3.85	0.56 ± 6.64	0.254
	Δ_12 week-baseline_	−1.44 ± 3.84 **	0.63 ± 7.60	0.142
Hip circumference (cm)	Baseline	102.59 ± 5.03	101.71 ± 4.83	0.438
	6 weeks	101.27 ± 4.67	101.41 ± 5.35	0.902
	12 weeks	101.47 ± 5.06	101.77 ± 4.79	0.786
	Δ_6 week-baseline_	−1.31 ± 1.92 **	−0.30 ± 3.79	0.150
	Δ_12 week-baseline_	−1.12 ± 2.69 **	0.06 ± 3.00	0.071
Waist-to-hip ratio	Baseline	0.87 ± 0.05	0.86 ± 0.09	0.493
	6 weeks	0.87 ± 0.06	0.87 ± 0.05	0.605
	12 weeks	0.87 ± 0.06	0.86 ± 0.06	0.890
	Δ_6 week-baseline_	0.00 ± 0.04	0.01 ± 0.07	0.688
	Δ_12 week-baseline_	0.00 ± 0.03	0.01 ± 0.08	0.485

Data are presented as mean ± standard deviation. * *p*-values were calculated using independent *t*-test. ** were represented as having *p*-values <0.05 in the paired *t*-test for comparison within-group differences.

**Table 4:** Effect of *Gelidium elegans* on body composition.

Variables		Test (*n* = 41)	Placebo (*n* = 37)	*p*-Value *
DEXA				
Total body mass	Baseline	72.78 ± 12.08	71.64 ± 10.61	0.662
(kg)	12 weeks	71.57 ± 11.90	72.10 ± 10.50	0.836
	Δ_12 week-baseline_	−1.21 ± 1.66 **	0.46 ± 1.19 **	<0.0001
Total fat mass	Baseline	19.66 ± 3.89	19.76 ± 4.23	0.919
(kg)	12 weeks	19.18 ± 3.80	20.84 ± 4.30	0.075
	Δ_12 week-baseline_	−0.48 ± 1.06 **	1.08 ± 0.75 **	<0.0001
Lean body mass	Baseline	50.68 ± 11.12	49.48 ± 10.51	0.626
(kg)	12 weeks	49.95 ± 10.91	48.85 ± 10.46	0.652
	Δ_12 week-baseline_	−0.73 ± 1.65 **	−0.63 ± 1.05**	0.743
Fat CT				
Visceral fat	Baseline	99.12 ± 43.75	93.97 ± 43.73	0.605
(cm^2^)	12 weeks	91.76 ± 38.95	95.42 ± 45.48	0.703
	Δ_12 week-baseline_	−7.36 ± 21.83 **	1.45 ± 13.53	0.034
Subcutaneous fat	Baseline	191.24 ± 56.72	198.49 ± 64.07	0.598
(cm^2^)	12 weeks	185.58 ± 54.96	210.12 ± 67.08	0.080
	Δ_12 week-baseline_	−5.67 ± 19.68	11.63 ± 21.49 **	0.0004
Total abdominal fat	Baseline	290.36 ± 81.59	292.46 ± 81.79	0.910
(cm^2^)	12 weeks	277.33 ± 75.03	305.54 ± 80.18	0.113
	Δ_12 week-baseline_	−13.03 ± 27.95 **	13.08 ± 26.51 **	<0.0001

Data are presented as mean ± standard deviation. * *p*-values were calculated using independent *t*-test. ** were represented as having *p*-values <0.05 in the paired t-test for comparison within-group differences.

**Table 5:** Fasting glucose and lipid profile.

Variables		Test (*n* = 41)	Placebo (*n* = 37)	*p*-Value *
Fasting glucose	Baseline	93.49 ± 10.38	93.49 ± 9.51	0.9995
(mg/dL)	6 weeks	95.34 ± 9.37	91.92 ± 8.23	0.092
	12 weeks	93.00 ± 10.33	94.38 ± 10.44	0.560
	Δ_6 week-baseline_	1.85 ± 8.66	–1.57 ± 9.28	0.096
	Δ_12 week-baseline_	–0.49 ± 8.18	0.89 ± 11.20	0.534
Fasting insulin	Baseline	8.45 ± 4.93	9.51 ± 5.62	0.377
(mIU/L)	6 weeks	9.27 ± 5.93	8.94 ± 4.83	0.791
	12 weeks	8.03 ± 4.04	9.65 ± 4.81	0.110
	Δ_6 week-baseline_	0.82 ± 3.51	–0.57 ± 5.51	0.194
	Δ_12 week-baseline_	–0.42 ± 4.65	0.14 ± 5.14	0.617
HOMA-IR	Baseline	1.98 ± 1.25	2.24 ± 1.45	0.396
	6 weeks	2.24 ± 1.56	2.08 ± 1.24	0.611
	12 weeks	1.88 ± 1.05	2.31 ± 1.31	0.116
	Δ_6 week-baseline_	0.26 ± 0.94	–0.17 ± 1.40	0.124
	Δ_12 week-baseline_	–0.10 ± 1.20	0.06 ± 1.31	0.567
HOMA-β	Baseline	94.52 ± 94.34	120.53 ± 70.79	0.176
	6 weeks	104.39 ± 56.21	113.28 ± 56.87	0.490
	12 weeks	104.83 ± 61.57	115.35 ± 59.39	0.446
	Δ_6 week-baseline_	9.87 ± 83.36	–7.25 ± 73.65	0.342
	Δ_12 week-baseline_	10.31 ± 91.36	–5.18 ± 76.32	0.422
hs-CRP	Baseline	1.36 ± 1.96	2.14 ± 5.79	0.442
(mg/L)	6 weeks	1.06 ± 1.25	1.61 ± 2.94	0.298
	12 weeks	1.06 ± 0.91	1.13 ± 0.98	0.722
	Δ_6 week-baseline_	–0.30 ± 2.10	–0.53 ± 6.46	0.839
	Δ_12 week-baseline_	–0.30 ± 1.79	–1.00 ± 5.75	0.482
Total cholesterol	Baseline	190.37 ± 28.98	190.03 ± 36.68	0.964
(mg/dL)	6 weeks	190.76 ± 26.02	196.22 ± 33.84	0.424
	12 weeks	191.51 ± 30.39	199.46 ± 36.02	0.294
	Δ_6 week-baseline_	0.39 ± 19.05	6.19 ± 16.47 **	0.157
	Δ_12 week-baseline_	1.15 ± 17.44	9.43 ± 20.94 **	0.061
LDL	Baseline	112.95 ± 27.99	112.81 ± 34.57	0.985
(mg/dL)	6 weeks	116.44 ± 26.48	118.73 ± 31.15	0.727
	12 weeks	114.27 ± 28.81	119.81 ± 32.60	0.428
	Δ_6 week-baseline_	3.49 ± 14.99	5.92 ± 17.13 **	0.507
	Δ_12 week-baseline_	1.32 ± 13.64	7.00 ± 21.06	0.168
HDL	Baseline	56.59 ± 10.97	57.19 ± 12.51	0.821
(mg/dL)	6 weeks	55.80 ± 11.12	56.78 ± 12.58	0.716
	12 weeks	56.66 ± 11.32	57.84 ± 12.73	0.666
	Δ_6 week-baseline_	–0.78 ± 7.12	–0.41 ± 5.36	0.795
	Δ_12 week-baseline_	0.07 ± 6.57	0.65 ± 7.04	0.710
Triglyceride	Baseline	125.22 ± 123.37	121.81 ± 83.08	0.886
(mg/dL)	6 weeks	112.41 ± 64.58	118.24 ± 65.10	0.693
	12 weeks	113.56 ± 87.94	129.30 ± 83.00	0.420
	Δ_6 week-baseline_	–12.80 ± 79.59	–3.57 ± 56.33	0.553
	Δ_12 week-baseline_	–11.66 ± 79.86	7.49 ± 49.92	0.204

Data are presented as mean ± standard deviation. * *p*-values were calculated using an independent *t*-test. HOMA-IR, homeostasis model analysis-insulin resistance; hs-CRP, high-sensitivity C-reactive protein; LDL, low-density lipoprotein; HDL, high-density lipoprotein. ** were represented as having *p*-values < 0.05 in the paired *t*-test for comparison within-group differences.
